# Editorial: Advancements and challenges in cost and resource allocation: 2022

**DOI:** 10.3389/frhs.2023.1254318

**Published:** 2023-07-20

**Authors:** Firdaus Hafidz, Chris Sampson

**Affiliations:** ^1^ Department of Health Policy and Management, Faculty of Medicine, Public Health, and Nursing, Universitas Gadjah Mada, Yogyakarta, Indonesia; ^2^ The Office of Health Economics, London, United Kingdom

**Keywords:** access to care, cost-effectiveness, economic evaluation, healthcare interventions, resource allocation

**Editorial on the Research Topic** Advancements and challenges in cost and resource allocation: 2022

## 1. Introduction

The efficient use of healthcare resources and assessments of cost-effectiveness are paramount in decision-making for health services. We launched a Research Topic exclusively for publications authored by members of the *Frontiers in Health Services* editorial team, with a view to collecting a variety of studies from 2022 that fall within the scope of our Section. The resulting Research Topic demonstrates the breadth of methods and topics that are relevant to *Cost and Resource Allocation*.

This editorial synthesises the collection of articles which delve into various facets of cost and resource allocation in healthcare. With meticulous research and analysis, the articles illuminate cost-effectiveness, capacity development needs, and barriers to accessing care across different healthcare interventions and services. Such research is a necessary input to effective evidence-based decision-making and health policy and seeks to influence a diverse set of stakeholders. The aim of this editorial is to provide a high-level summary of the key findings from these articles, underscoring their importance in enhancing our understanding of efficient resource allocation in healthcare.

## 2. Economic evaluation: a focus on infants and children

Part of this Research Topic reports on economic evaluations, the principle tool in determining the cost-effectiveness and efficiency of healthcare interventions. King et al. performed a cost-effectiveness analysis of heart rate characteristics (HRC) monitoring in very low birth weight (VLBW) infants, using data from a clinical trial. The study concluded that HRC monitoring was cost-effective in enhancing survival outcomes, saving lives with efficient use of resources. It is notable that the researchers use data from a trial published over a decade ago. The findings from this study have practical implications for healthcare providers and policymakers in contexts where HRC monitoring is already adopted, as well as those settings where decision-makers may be considering its adoption.

In a similar vein, Pace et al. examined the cost-effectiveness of a tele-resuscitation intervention in pediatric emergency departments. The study found the intervention to be cost-effective, indicating its potential to optimise healthcare delivery and resource allocation. The research employed interviews to understand the broader cost implications of healthcare, including out-of-pocket payments and productivity losses, which are important considerations in the evaluation of telehealth interventions. These findings underscore the importance of considering telemedicine interventions as possible solutions to enhance access to specialised care while managing costs effectively.

## 3. Access to care: tackling barriers and enhancing capacity

Access to care and capacity development are vital components of resource allocation in healthcare. Two studies in this Research Topic provide valuable insights into these areas. Hanney et al. explored the perceived barriers to accessing physical therapy services among individuals with low back pain in Florida. The study identified factors such as proximity to physical therapy clinics, availability of childcare, session duration, session frequency, and individual activity levels as significant barriers. These findings have important policy implications, suggesting that addressing these barriers through targeted interventions and policy changes can enhance access to physical therapy services, reduce healthcare disparities, and optimise resource allocation.

Ward et al. investigated the capacity development needs of a national prostate cancer service in Scotland. The study identified several barriers to capacity development, including a lack of shared understanding of the quality of care between policymakers and healthcare professionals, a lack of leadership of service developments at national and regional levels, and difficulties in drawing on other capacities to support the service. The study also highlighted the need for cohesive working and efficient training for nurse specialists to develop capacity. These findings underscore the importance of addressing these barriers and facilitating capacity development to enhance the ability of healthcare services to meet demand, improve the quality of care, and optimise resource allocation.

## 4. Bridging economic evaluation and access to healthcare

It is important for research to characterise the link between economic evaluation and access to healthcare. Economic evaluations provide crucial information on the cost-effectiveness of interventions, enabling policymakers to make informed decisions regarding resource allocation. By identifying interventions that are not only clinically effective but also cost-effective, policymakers can prioritise the allocation of resources to maximise health outcomes within the available budget. Robust evidence on cost-effectiveness can support buy-in from stakeholders that help to ensure access to care.

However, cost-effectiveness alone does not ensure equitable access to healthcare services. Barriers to access, as identified in the study by Hanney et al., can prevent individuals from receiving necessary care, even when the intervention is deemed cost-effective. Therefore, it is essential to address these barriers in conjunction with economic evidence. Policymakers should focus on developing strategies that enhance access to cost-effective interventions, ensuring that all individuals, regardless of their socioeconomic background, have equal opportunities to benefit from them.

The collection of articles in this editorial offers valuable insights into cost and resource allocation in healthcare, with a focus on economic evaluations and access to care. The findings have practical implications for healthcare providers, policymakers, and stakeholders, guiding decision-making processes and promoting efficient allocation of resources. By integrating cost-effectiveness considerations and addressing barriers to access, healthcare systems can enhance the delivery of cost-effective interventions and ensure equitable access to care. Future opportunities lie in exploring long-term cost-effectiveness, refining financing mechanisms, and leveraging technology to optimise resource allocation. Continued research and collaboration in this field will contribute to building sustainable healthcare systems that deliver high-quality care to all.

